# The Roles of Oxytocin and CD38 in Social or Parental Behaviors

**DOI:** 10.3389/fnins.2012.00182

**Published:** 2013-01-11

**Authors:** Olga Lopatina, Alena Inzhutova, Alla B. Salmina, Haruhiro Higashida

**Affiliations:** ^1^Department of Biochemistry, Medical, Pharmaceutical and Toxicological Chemistry, Krasnoyarsk State Medical UniversityKrasnoyarsk, Russia; ^2^Research Center for Child Mental Development, Kanazawa UniversityKanazawa, Japan

**Keywords:** oxytocin, social behavior, social experience, parental care, CD38/ADP-ribosyl cyclase activity

## Abstract

The nine amino acid peptide oxytocin (OXT) has been directly associated with different types of behavioral reactions. The formation and maintenance of social relationships in youth and middle age are important components of human mental health. A deficit in healthy behavioral formation leads to social isolation and limitation of well-being. Mice are social animals and are therefore useful for investigating the neurobiological mechanisms of cognitive process control, including the development of social relationships and social skills. Studies in mice may broaden our understanding of the human condition. The multifunctional protein CD38/ADP-ribosyl cyclase is highly expressed in the brain, plays an important role in central OXT release, and regulates social memory. In this review article, we discuss the mechanisms of social behavior affected by the dysregulation of brain OXT function as a consequence of a lack of CD38. OXT bound to OXT receptors initiates autoregulatory positive feedback of OXT release in the hypothalamus and posterior pituitary. OXT bio-behavioral positive feedback is usually implicated in female reproductive systems, but can also be observed in social behavior. Exogenous stimuli (OXT treatment *in vitro*, OXT intravenous or intraventricular administration, and nasal OXT delivery) initiate activation of OXT neurons via PKC-CD38/ADP-ribosyl cyclase cascades and result in the modulation of social behavior in humans and mice. Based on these findings, we reviewed the functions of OXT and its properties with respect to the development of therapies for human social behavior impairments in psychological diseases. In addition, preliminary studies of continuous nasal OXT administration on subjects with autism spectrum disorders are described.

## Introduction

The nine amino acid peptide oxytocin (OXT) plays a dual role with peripheral and central effects in the regulation of many physiological and pathophysiological processes, including penile erection and ejaculation (Uckert et al., 2003; Vignozzi et al., 2004; Thackare et al., 2006), pregnancy and uterine contractions, milk ejection (Kendrick and Keverne, 1992; Keverne and Kendrick, 1992), osteoporosis (Elabd et al., 2008; Tamma et al., 2009), diabetes (Björkstrand et al., 1996; Gutkowska et al., 2009), cancer (Cassoni et al., 1996, 2002), the functioning of the cardiovascular system (Jankowski et al., 1998, 2012; Petersson and Uvnäs-Moberg, 2008), sexual activity (Pedersen and Boccia, 2002, 2006), pain modulation (Yang, 1994; Condés-Lara et al., 2005), stress, trust (Kosfeld et al., 2005; Hoge et al., 2012), anxiety (McCarthy et al., 1996; Heinrichs and Domes, 2008; Campbell, 2010), social interaction and bonding (mother-infant bonding or pair bonding) (Popik et al., 1992; Benelli et al., 1995; Insel, 1997, 2010; Kendrick, 2000; Young et al., 2001; Wang and Aragona, 2004; Young and Wang, 2004; Ebstein et al., 2009, 2011; Meyer-Lindenberg et al., 2011), and parental care (Modney and Hatton, 1994; Kendrick et al., 1997; Meaney, 2001; Fleming et al., 2002; Feldman and Eidelman, 2007; Feldman et al., 2011; Liu et al., 2012b). OXT is important for the processing or retention of direct and indirect social information (Ferguson et al., 2001; Kavaliers et al., 2006; Modi and Young, 2012). The specific pattern of OXT secretion is related to the characteristics of behavioral reactions (Higashida et al., 2010, 2011, 2012a,b; Salmina et al., 2010).

With respect to OXT, we reported that CD38, a type II transmembrane protein, which controls leukemia malignancy in blood cells (Malavasi et al., 2008), is expressed in the brain and required for OXT secretion in mice (Jin et al., 2007). CD38 possesses ADP-ribosyl cyclase activity (Lee, 2012) that produces cyclic ADP-ribose (cADPR) from β-NAD^+^, which is an abundant substrate in the brain. cADPR is proposed as an intracellular second messenger, in that cADPR functions as a cofactor for Ca^2+^ mobilization through Ca^2+^-permeable channels (Ca^2+^-induced Ca^2+^-release, CICR) from ryanodine-sensitive Ca^2+^ pools, resulting in increases of cytosolic free Ca^2+^ concentrations ([Ca^2+^]_i_). Therefore, it is postulated that some cellular events such as secretion or cell migration depend on the formation of cADPR.

In this review, we discuss recent research on the multiple functions of OXT and CD38 in social and parental behaviors. We propose cellular and systemic mechanisms for OXT and CD38 in social and parental behaviors, and we draw a schematic model of the signaling mechanism in a comprehensive manner. Finally, we focus on both single nucleotide polymorphisms (SNPs) of the human CD38 gene in relation to autism spectrum disorders (ASDs) and repetitive treatment of ASD patients with nasal administration of OXT.

## Oxytocin and Social Relationships in Humans

Oxytocin is involved in different types of mammalian social behavior from rodents to humans in both sexes (Striepens et al., 2011). Social memory, as part of social behavior, is based on the ability to recognize conspecific forms (kin, mates, offspring, allies, and enemies) and is crucial for social life (DeBruine et al., 2008). In humans, faces provide important information about identity. OXT improves an individual’s ability to produce normative ratings of others’ emotions based on pictures of the eye regions of healthy adults (Domes et al., 2007). The blood OXT level is correlated with feelings of attachment (Tops et al., 2007; Campbell, 2008; Marazziti et al., 2009; Strathearn et al., 2009). OXT levels in the brain are increased in individuals with higher constructive approaches (Dai et al., 2012) compared to those with attachment avoidance (De Dreu, 2012). OXT may play an important health-promoting role in positive couple interactions (Ditzen et al., 2009).

The physiological functions of OXT in the regulation of mental health are confirmed by numerous studies of neuropsychiatric disorders. Individuals with obsessive compulsive disorder (OCD), ASDs, eating disorders, addiction, schizophrenia, and posttraumatic stress disorder (PTSD) show dysregulation of OXT levels (Leckman et al., 1994; Frasch et al., 1995; Marazziti and Cassano, 2003; Marroni et al., 2007; Meinlschmidt and Heim, 2008; Ishak et al., 2011). Trauma (Pierrehumbert et al., 2010) and PTSD (Marazziti and Cassano, 2003), as well as depression in women (Cyranowski et al., 2008), are associated with high pulsatile OXT levels, and very low OXT levels are associated with schizophrenia and ASD (Goldman et al., 2008; Kéri et al., 2009; Yamasue et al., 2009; Higashida et al., 2012b; Modi and Young, 2012).

The formation and maintenance of social relationships in youth and middle age are essential components of human mental health. Gaining an understanding of the neural, humoral, and genetic factors that regulate social behavior is crucial for human well-being (Kendrick, 2006). A deficit in healthy behavioral formation (ASD, schizophrenia, or social phobia) leads to social isolation. Thus, researchers need to understand the molecular mechanisms that sustain the establishment and modulation of relationships between individuals, especially in the context of treatment and drug therapy for patients. At present, little is known about the molecular mechanisms of OXT secretion in the context of social behavior in humans (Meyer-Lindenberg et al., 2011). Therefore, adequate animal models of OXT-mediated behavioral reactions are urgently required. Mice are social animals and are useful as models for investigating the neurobiological mechanisms of cognitive process control, which lead to the development of social relationships and skills. Studies in these animals may broaden our understanding of the human condition (Baker, 2011). A number of studies on the neurobiological bases of social behavior with mouse models have been performed; these studies were enriched with genetic technology in the form of gene “knockout” model mice.

## Oxytocin, Social Memory, and CD38 in Rodents

Male mice deficient in the gene encoding OXT (oxytocin knockout mice, *Oxt^−/−^*) displayed deficits in social recognition (Ferguson et al., 2000; Modi and Young, 2012) that could be reversed by intracerebroventricular infusion of OXT, and an infusion of an OXT antagonist inhibited social recognition in wild-type mice (Ferguson et al., 2001). This deficit was specific for social recognition: *Oxt^−/−^* mice showed no impairments in other forms of learning or olfactory sensitivity and discrimination (Ferguson et al., 2000). Similar impairments in social recognition occurred in female *Oxt^−/−^* mice (Choleris et al., 2003). *Oxt^−/−^* mice showed increased anxiety and stress responses to psychogenic and certain physiological stimuli (Mantella et al., 2003; Amico et al., 2004). OXT receptor (OXTR) knockout mice (*Oxtr^−/−^*) emitted fewer ultrasonic vocalizations (USV), had higher levels of aggression, and showed social memory impairment (the latter of which could be abolished by OXT administration) compared to wild-type littermates (Takayanagi et al., 2005; Crawley et al., 2007; Macbeth et al., 2009; Sala et al., 2011).

However, social behavioral deficits could be caused not only by the lack of OXT and OXTR genes, but also by disturbance of the molecular mechanism involved in the cascade of OXT release. Deletion of the *Cd38* gene in mice leads to deficits in social behavior due to abnormal central OXT secretion (Jin et al., 2007; Liu et al., 2008). CD38/ADP-ribosyl cyclase is a trifunctional enzyme, which is involved in the catalysis of cADPR from NAD^+^. This enzyme regulates intracellular calcium levels and is also responsible for hydrolysis of this molecule, as well as the total NAD^+^-glycohydrolase activity (Howard et al., 1993; Lee and Aarhus, 1995; Magni et al., 2004; Salmina et al., 2010; Lee, 2012). CD38 is expressed in murine (Ceni et al., 2003; Jin et al., 2007) and human brains (Mizuguchi et al., 1995; Munesue et al., 2010) and accounts for the majority of ADP-ribosyl cyclase activity *in vitro* (Malavasi et al., 2008; Lee, 2012). Cyclase activity corresponding to CD38 was detected in the brain during early embryonic mouse development, and the postnatal activity was enhanced until adult stages (Ceni et al., 2003, 2006).

## Oxytocin and Cyclic ADP-Ribose

**Figure 1:**
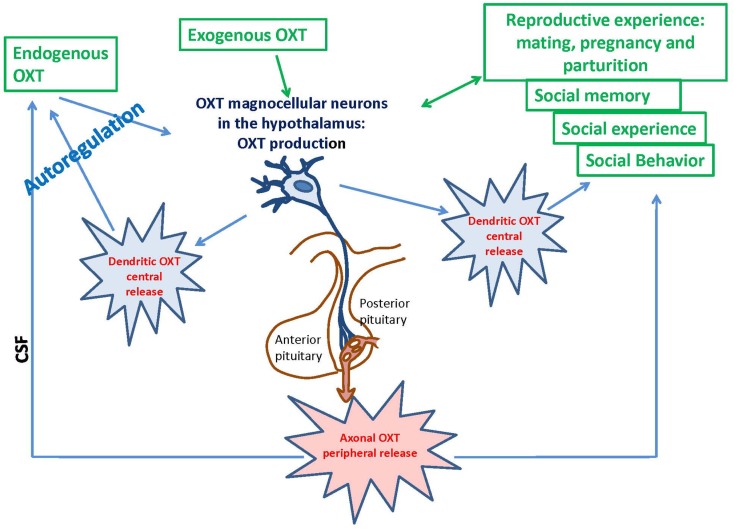
**Deletion of the *Cd38* gene affects ADP-ribosyl cyclase activity, oxytocin release, and social behavior in male mice throughout their lifespans: significance of weaning**.

Oxytocin is mainly synthesized in the paraventricular hypothalamic nucleus (PVN) and supraoptic nucleus (SON), stored in Herring bodies and released into systemic circulation from the posterior pituitary (Oliver and Schäfer, 1895; Richard et al., 1991; Figure 1). ADP-ribosyl cyclase activity was demonstrated in the hypothalamus and posterior pituitary of the mouse brain; the activity in the hypothalamus was dominant (Jin et al., 2007). *Cd38* gene knockout mice (*Cd38^−/−^*) show impairment of glucose-induced increases in cADPR, Ca^2+^ concentration, and insulin secretion in pancreatic β-cells and the absence of Ca^2+^ oscillations in T cells (Takasawa et al., 1998; Kim et al., 2008). The lack of CD38 results in decreased ADP-ribosyl cyclase activity, lower levels of cADPR formation, and dysfunction of CICR; these deficiencies lead to alterations in OXT secretion in the hypothalamus and pituitary (Jin et al., 2007; Lopatina et al., 2010). This CD38-dependent secretion was specific to OXT but not to other transmitters, such as dopamine in the striatum or vasopressin in the hypothalamus (Jin et al., 2007). Other types of voltage-dependent Ca^2+^ channels are also involved in oxytocinergic neurons (Tobin et al., 2011). Recently, we showed that OXT release is also sensitive to hyperthermia and Ca^2+^ influx via TRPM2 channels (Amina et al., 2010; Liu et al., 2012a).

## Signal Transduction and CD38 in Rodents

**Figure 2:**
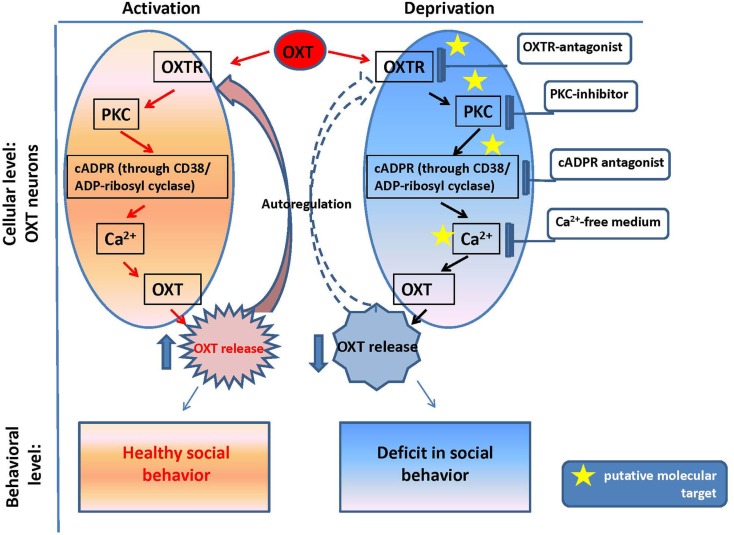
**Oxytocin positive feedback control and oxytocin signaling in psychophysiological development**.

The reproductive experience, rodent pup stimulation (sucking and olfactory signals), neurotransmitters, and hormones responsible for establishing parental behavior can activate many receptor complexes. For example, social (paternal) experience coincides with the efficiency of OXTR binding (Parker et al., 2001; De Jong et al., 2009). Receptor stimulation leads to the elevation of neuronal calcium levels and activation of the protein kinase C (PKC; Fleming et al., 1999). OXT is released from the axons of hypothalamic neurons, interacts with OXTR, and stimulates production of inositol 1,4,5-trisphosphate (IP3) and diacylglycerol (DAG) through the actions of phospholipase C (PLC; Gimpl and Fahrenholz, 2001) and PKC (Figure 2). Thus, this PLC- and IP_3_-dependent Ca^2+^ signaling pathway may function in the mechanism of autoregulation of OXT release (Lambert et al., 1994), i.e., the direct action of OXT on OXT neurons mediated by OXTR. The positive feedback mechanism of OXT release plays a critical and physiological role in causing uterine contractions during labor and milk release during breastfeeding in rodents (Moos et al., 1984; Neumann et al., 1994, 1996).

In rats, the binding of OXT to OXTR causes biochemical and transcriptional changes that account for the immediate and long-term neuromodulatory effects of OXT (Erwin et al., 2011). Blocking OXTR reduces lactation-related behaviors (Pedersen and Boccia, 2003; Bosch and Neumann, 2008), increases anxiety-related behaviors (Bosch and Neumann, 2008), and affects maternal offensive and defensive behaviors (Bosch et al., 2005; Febo et al., 2009). An *in vitro* study showed that OXT stimulates its own release from tissue blocks containing both SON and PVN (Moos et al., 1984). Many neuronal OXT responses are important for particular behavioral or physiological functions. Elucidating the signal transduction mechanisms mediating the effects of released OXT on cellular characteristics may reveal the principles of critical autoregulation for the functioning of the mammalian brain (Dayanithi et al., 2000; Landgraf and Neumann, 2004).

We were interested in the mechanism by which CD38/ADP-ribosyl cyclase is activated after OXTR stimulation in the hypothalamus (which leads to secretion of OXT) and how this mechanism is related to social recognition or social behavior in the *Cd38^−/−^* strain (Jin et al., 2007; Liu et al., 2008).

## Oxytocin and Cytosolic Calcium in the Hypothalamus

Our *in vitro* experiments with the hypothalamus and posterior pituitary of adult male mice (Lopatina et al., 2010) indicated the involvement of the CD38/Cyclic ADP-ribosyl systems in the autoregulation of OXT secretion. The maximum increase in ADP-ribosyl cyclase activity (in crude membranes prepared from the hypothalamus and posterior pituitary of adult male mice in response to 10 nM OXT) was 1.6-fold higher than the pre-exposure levels in the hypothalamus, while the activity was increased by 2.8-fold in response to 10 pM OXT in the pituitary (Lopatina et al., 2010). Simultaneous application of vasotocin, an OXTR antagonist, significantly inhibited the OXT-induced increase in ADP-ribosyl cyclase activity. Intracellular cADPR levels increased during incubation with OXT for 5 min in a dose-dependent manner. ADP-ribosyl cyclase was also activated by kinases via the OXT signaling pathway, which was sensitive to 5 nM staurosporine (a non-selective inhibitor of protein kinases) and 100 nM calphostin C (a specific PKC inhibitor) in both tissues. The results confirmed that OXT-mediated OXT release in male mice, and this process was dependent on both cADPR and Ca^2+^, which are mediated by PKC (Figure 2). The OXT-induced reactions in the signal cascade were sensitive to PKC inhibitors.

The OXT release was observed in tissue blocks acutely isolated from the mouse hypothalamus, in which the nerve terminals of the oxytocinergic neurons were not present (Jin et al., 2007). Thus, OXT secretion observed in such conditions is caused by cell soma, recurrent axons, and axonal swellings. This somato-axonal OXT release was closely correlated with intracellular Ca^2+^ dynamics. More importantly, the endoplasmic reticulum Ca^2+^ stores play a major role in Ca^2+^ homeostasis in identified OXT neurons because no release was detected in depletion of stored Ca^2+^ under the Ca^2+^-free condition (Higashida et al., 2010; Salmina et al., 2010). Because a Ca^2+^-free medium is assumed to block synaptic transmission, the above results suggest a direct action of OXT on OXT neurons (Figure 2).

The OXT-induced Ca^2+^ elevation is due to cADPR-induced Ca^2+^ release from intracellular stores mediated by ryanodine receptors in a PKC-dependent manner, followed by Ca^2+^ mobilization due to activation of the IP_3_ receptors, which was not sensitive to PKC in the mouse hypothalamus (Lopatina et al., 2011). PKC is involved in the stimulation of ADP-ribosyl cyclase activity-mediated [Ca^2+^]_i_ increase, and facilitation of OXT release. Autoregulation is usually attributed to the female reproductive system. Fleming et al. (1999) previously demonstrated that maternal experience initially stimulates enhanced PKC synthesis and activation of c-*fos* gene expression in the maternal system [e.g., medial preoptic area (MPOA)]. Reproduction-related stimuli cause positive feedback release of OXT within the brains of lactating rats (Brunton and Russell, 2010). Our data on parental behaviors and social recognition, and the findings of our *in vitro* study indicate that social (reproductive) experience activates OXT neurons, increases hypothalamic CD38/ADP ribosyl cyclase activity, stimulates OXT release from the axons and potentially from the dendrites, and induces OXT autoregulation (Lopatina et al., 2011; Higashida et al., 2012b). These observations together indicate that positive feedback of PKC- and cADPR-dependent OXT release in the hypothalamus and pituitary is important for correct and efficient social conduct in relation to social stimulation (Figure 2). OXT initiates activation of OXTR+ neurons through the PKC-CD38/ADP-ribosyl cyclase cascade, thus leading to modulation of social behavior in mice. Such a mechanism may also be an important component of human social behavior adjustment to external stimuli (Figure 1).

## Oxytocin Levels in CD38 Knockout Mice

**Figure 3:**
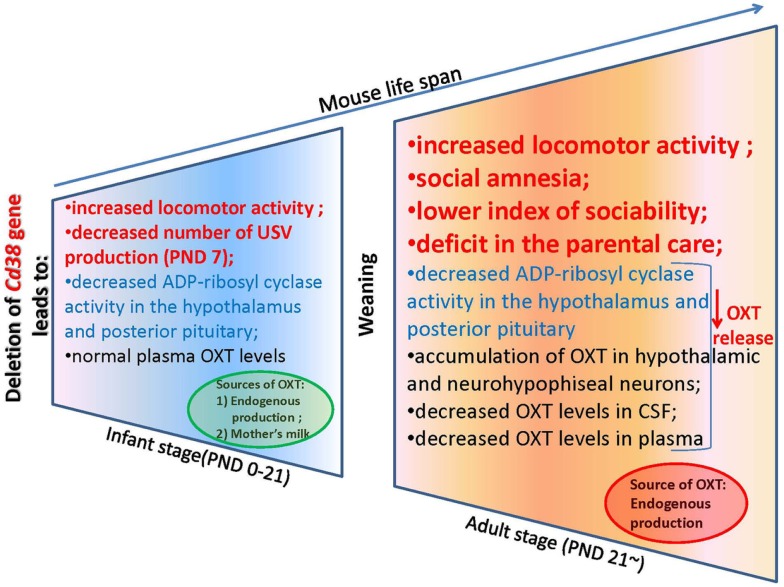
**An oxytocin release feedback loop and potential molecular targets for pharmacotherapy of alterations in social behavior**.

To explain this tendency, we measured the plasma OXT levels in the CD38 knockout mice (Liu et al., 2008). Interestingly, the plasma OXT levels were comparable in both genotypes during the first 3 weeks after birth until weaning; this was followed by a significant reduction of plasma OXT levels in *Cd38^−/−^* mice after the weaning period (>3 weeks). In *Cd38^−/−^* mice, ADP-ribosyl cyclase activity was markedly lower in the hypothalamus and pituitary from the first postnatal day and was consistently lower thereafter until the adult stage in comparison with *Cd38*^+*/*+^ mice (Figure 3). The reduced severity of behavioral abnormalities in *Cd38^−/−^* pups was due to partial compensation by high levels of plasma OXT. Therefore, the weaning time in *Cd38^−/−^* mice seems to be a critical period for distinguishing different plasma OXT levels as the mice transition from the infant to the adult stage. We speculated that *Cd38^−/−^* pups take in OXT from the dams’ milk, which helps them recover from their own OXT secretion deficits (Higashida et al., 2010). We found that OXT was abundant in the mammary gland tissue and milk of lactating dams of both genotypes. Milk OXT may be transported into the bloodstream.

This supports the suggestion that maternal behavior is directed at infant care not only by sustaining protection and nurturing, but also by permitting a longer period of brain development after birth (Pedersen, 1999; Feldman and Eidelman, 2007). As expected, human studies showed that plasma and salivary OXT levels in mothers are associated with parent and child’s social engagement, affect synchrony, and positive communicative sequences between the parent and child (Feldman and Eidelman, 2003; Feldman et al., 2004, 2010, 2011).

## CD38 and Oxytocin Secretion in Mice

The plasma and cerebrospinal fluid (CSF) OXT levels were significantly lower in *Cd38^−/−^* mice than *Cd38*^+*/*+^ mice, but the OXT levels were elevated in the hypothalamus and pituitary, in comparison with wild-type mice. These data clearly demonstrate normal OXT production and packaging into vesicles in the hypothalamic neurons and posterior pituitary nerve endings in *Cd38^−/−^* mice, but altered OXT release into the brain and bloodstream (Jin et al., 2007; Higashida et al., 2012b). Indeed, the behavioral phenotypes of *Cd38^−/−^* mice could be normalized by even a single subcutaneous OXT injection and also by infusion of a virus carrying the human *CD38* gene into the third ventricle of knockout mice; this result indicates the requirement of CD38-dependent OXT secretion for development of special types of social behavior (Jin et al., 2007; Higashida et al., 2010; Salmina et al., 2010; Figure 2).

We also identified significant abnormalities in maternal nurturing behavior in *Cd38^−/−^* postpartum mice under stressful conditions, such as pup–dam separation (Jin et al., 2007). Female *Cd38^−/−^* mice displayed disrupted maternal behavior in the retrieval test. Wild-type dams retrieved all five test pups very quickly and directly to the nest arena, whereas *Cd38^−/−^* dams took a significantly longer time to begin retrieval, moved around continuously, and often dropped the pups during retrieval (suggesting memory loss of the way to the nest), resulting in the pups becoming scattered in different places. However, after reunion, *Cd38^−/−^* dams fed the pups sufficiently for them to grow to the same weight as the controls (Jin et al., 2007; Lopatina et al., 2011; Higashida et al., 2012a).

## Social Behaviors in CD38 Knockout Mice

We now describe the social behavior in *Cd38^−/−^* mice in relation to the brain and plasma OXT levels in the context of CD38/ADP-ribosyl cyclase activity-dependent mechanisms of OXT secretion. We especially focus on infant male behavior, social skills in adult males, and parental (maternal and paternal) behavior in *Cd38^−/−^* mice (Figure 3). In addition, we summarize the ADP-ribosyl cyclase/cADPR-controlled [Ca^2+^]_i_ signaling involved in the autoregulatory positive feedback of OXT release in the hypothalamus and posterior pituitary, resulting in special types of social behavior.

*Cd38^−/−^* mice grew well and showed the same weight gain as wild-type (*Cd38*^+*/*+^) mice (Jin et al., 2007; Liu et al., 2008). Alterations in locomotor activity and exploration are important consequences of the paradigms used to study specific processes, such as learning, memory, and anxiety. The individual and group locomotor activities (induced by separation stress from the dam) were significantly higher in 7-day-old *Cd38^−/−^* mouse pups than in *Cd38*^+*/*+^ controls (Liu et al., 2008; Figure 1). Locomotor abnormalities are associated with human psychiatric diseases (Gil-Bea et al., 2007; Touma et al., 2008; Silverman et al., 2011; Won et al., 2011); these diseases are difficult to model in rodents because of the variability of symptoms and the absence of verbal communication (Onaivi et al., 2011). Nevertheless, a number of relevant behavioral and social changes have been documented in transgenic mouse models of neurodevelopmental disorders (Branchi et al., 2001; Brooks et al., 2005; Crawley, 2007; Moy et al., 2007, 2008; Wöhr et al., 2011).

## Oxytocin and Reproduction Experience in Mice

The search for the mechanisms that control the transmission of the OXT bio-behavioral feedback loop indicated that social experience may modulate brain plasticity (Insel and Young, 2001), and OXT production and functions are based on behavioral and neural changing mechanisms as well as on genetic mechanisms (Modney and Hatton, 1994; Fleming et al., 1999). One of the constituent parts of social experience is the reproductive experience (including mating, pregnancy, and parturition; Figure 1). The reproductive experience is also an important factor for the expression of maternal behavior, and additional parenting experience is necessary to confer induction of parental maternal behavior (Okabe et al., 2010; Liu et al., 2012b; Nagasawa et al., 2012). OXT-induced long-term potentiation is affected by mothering (Tomizawa et al., 2003). The initial (primiparity and mothering) reproductive experience results in behavioral, hormonal, and neural changes in the mother that markedly alter subsequent reproductive experiences (Pawluski et al., 2006). Therefore, we examined the positive effect on reproductive experience in parental behavior by multiparous *Cd38^−/−^* dams. They retrieved pups faster than primiparous *Cd38^−/−^* mice, whereas there were no significant differences between primiparous and multiparous *Cd38*^+*/*+^ dams in the retrieval test (Lopatina et al., 2011). The plasma OXT levels were significantly increased in multiparous dams compared to primiparous dams of both genotypes. In addition, OXT levels in the hypothalamus and pituitary were lower in *Cd38^−/−^* dams than wild-type controls because OXT is released into the brain and blood in experienced mice. ADP-ribosyl cyclase activity in the hypothalamus, but not in the pituitary, was slightly increased in *Cd38*^+*/*+^ dams. Thus, mouse maternal OXT is related to the reproductive experience and positive maternal behavior (Figure 3). Whether this mechanism is significant for human maternal behavior remains to be elucidated.

Associations between peripheral OXT and parenting were also found in fathers, suggesting that OXT neuronal pathways may be activated through the provision of paternal care (Feldman et al., 2010, 2011; Liu et al., 2012b). The experiments on the paternal behavior in mice showed that only 40% of first-time *Cd38*^+*/*+^ sires displayed paternal care in the retrieval test (Lopatina et al., 2011). Both first- and second-time *Cd38^−/−^* sires showed only 10% retrieval behavior. The time required to retrieve five pups to the nest was shorter for second-time *Cd38*^+*/*+^ sires, and this time was associated with increased hypothalamic ADP-ribosyl cyclase activity. Induction of ADP-ribosyl cyclase activity leads to stimulation of OXT release and elevated plasma OXT levels as observed in dams. Therefore, the reproductive experience improves parental behavior, especially in *Cd38^−/−^* dams, suggesting the involvement of OXT systems in reproductive experience-mediated remodeling of the neuroendocrine system.

## Social Information Transmission in Mice

In mammals, transmission of social information is critical for the establishment of all aspects of social behavior, sociability, and sociality: humans use language, while mice have USV, which may be a measure of social communication in mice (Crawley, 2004). Studies have used pup vocalizations as a sensitive behavioral endpoint (Iijima and Chaki, 2005; Scattoni et al., 2008). One standard test for vocalization in mice is the ultrasonic distress call of pups separated from the dam or removed from the nest (Winslow et al., 2000; Branchi et al., 2001; Shu et al., 2005). Mouse pups emit isolation-induced USVs that have the characteristics of songs, which consist of several different syllable types and the temporal sequencing includes the utterance of repeated phrases. The isolation-induced USVs are emitted by both *Cd38*^+/+^ and *Cd38^−/−^* pups and have the same frequency (∼70 kHz) and duration (∼60 ms). The number of USVs was significantly (1.6-fold) lower in *Cd38^−/−^* pups than in *Cd38*^+*/*+^ pups. Reduced USVs in mice are a useful parameter relevant to the second diagnostic symptom of autism, impaired communication (Klin et al., 2007; Scattoni et al., 2008). Our findings in *Cd38^−/−^* mice are very similar to the communicative alterations found in *Oxt^−/−^* and *Oxtr^−/−^* mice (Nishimori et al., 1996; Ferguson et al., 2000; Winslow et al., 2000; Takayanagi et al., 2005; Crawley et al., 2007). However, *Cd38^−/−^* mice have smoother patterns of behavioral expression than mice lacking the *Oxt* or *Oxtr* genes.

## Social Responses and CD38 in Mice

The formation and maintenance of social relationships are complex processes that involve several stages of information processing in the human brain. Investigations of social behavior in animals generally focus on a single level of processing at a given time point (Lim and Young, 2006). However, social recognition is necessary for the establishment of social bonds between individuals. Understanding the neurobiological bases of social recognition and the use of social information transmission in mice can allow translation of the proximal mechanisms of sociality to humans (Tang-Martinez, 2003; Choleris et al., 2004). Individual recognition can be operationally defined as unique modifications; an animal behaves toward another animal by relying on past experiences that are specific to individuals (Gheusi et al., 1994). The *Cd38* gene deficit is associated with social amnesia in social recognition tasks (Ferguson et al., 2000; Choleris et al., 2004), which detect the natural propensity of mice to investigate an intruder mouse that is presented repeatedly. In normal behavior, the social response of the resident mouse declines to very low levels (habituation). *Cd38^−/−^* males did not habituate to intruder females after repeated encounters and displayed sustained high levels of investigation at all encounters with the same female, whereas *Cd38*^+*/*+^ male mice exhibited a significant decline in the investigation time and positive social memory (Jin et al., 2007; Higashida et al., 2012b). This amnesia resembles the memory deficit observed in *Oxt^−/−^* and *Oxtr^−/−^* mice (Ferguson et al., 2000; Takayanagi et al., 2005). A single subcutaneous injection of OXT rescued the social memory deficits of *Cd38^−/−^* mice because OXT may enter the brain, probably due to the blood–brain barrier (BBB) permeability for OXT or some other pathways (McEwen, 2004; Bartz and Hollander, 2006; Hollander et al., 2007; Jin et al., 2007; Churchland and Winkielman, 2012). Social and individual recognition facilitates social interactions in group life and is considered to be one of the key evolutionary underpinnings of sociality (Altizer et al., 2003; Kavaliers et al., 2005; Choleris et al., 2009; Figure 1).

## Oxytocin, CD38, and Autism Spectrum Disorders

A recent series of studies in humans showed that nasal infusion of OXT increases trust (Kosfeld et al., 2005; Baumgartner et al., 2008), mindreading (Domes et al., 2007), and generosity (Zak et al., 2007), indicating an important role of OXT in human social behavior (MacDonald and MacDonald, 2010; MacDonald et al., 2011). Furthermore, OXT reduces repetitive behavior in adults with autism and Asperger’s disorder (Hollander et al., 2003).

Studies have reported associations between parental and infant OXT levels with the degree of contingent parenting (Feldman and Eidelman, 2003, 2007; Feldman et al., 2004). Maternal postpartum behavior has long-term effects on infants’ cognitive, neurobehavioral, and social-emotional growth. Mother-infant touch and contact stimulate OXT release (Matthiesen et al., 2001). OXT and CD38 are related to higher levels of parental care and longer episodes of gaze synchrony with infants (Feldman et al., 2012). Maternal OXT is related to sensitive and emotional behavior (Gordon et al., 2010; Strathearn et al., 2012) and an increased blood oxygenation-level dependent (BOLD) functional magnetic resonance imaging (fMRI) response to infant stimuli in brain areas rich with OXTR (Strathearn et al., 2009). Paternal OXT is correlated with tactile stimulation and exploratory play with tasks oriented toward their infants (Gordon et al., 2010; Weisman et al., 2012). Mothers and fathers who provided high levels of tactile contact to their infants showed an increase in salivary OXT following parent–infant interactions, but no increase was observed among parents who provided low tactile contact (Feldman et al., 2010). In a similar way, high and low licking-and-grooming patterns of rat and mouse dams have differential impacts on OXT expression. In humans, there is a general consensus that both prenatal and postpartum OXT enhance the formation of close bonds with the infant and reduce maternal stress reactivity (Nelson and Panksepp, 1998; Neumann, 2008; Campbell, 2010). OXT inhalation increased the fathers’ responsiveness to their toddlers, particularly in the father-specific pattern (Naber et al., 2010; Weisman et al., 2012). Variations in the OXTR gene were related to the degree of maternal sensitivity to OXT (Bakermans-Kranenburg and van Ijzendoorn, 2008; Feldman et al., 2010). The importance of SNPs of OXTR has been discussed in relation to ASDs (Ebstein et al., 2010; Insel, 2010). OXT activates neural circuitries related to empathy in women exposed to the crying of an infant (Riem et al., 2011). The central and peripheral OXT measurements revealed meaningful differences in parenting behavior in humans, similar to the roles in other mammals. The matching of rodent and human studies is valuable for translational research in this field of medicine.

*CD38* mRNA is expressed in many different regions in the human brain, including the hypothalamus, where CD38 colocalizes with oxytocinergic neuronal structures (Munesue et al., 2010). OXT plasma levels are lower in ASD patients than in individuals without this disorder (Modahl et al., 1998; Munesue et al., 2010). A mutation in the *CD38* gene is associated with ASD and lower OXT levels (Munesue et al., 2010). *CD38* expression in human lymphoblastoid cell (LBC) lines obtained from subjects with ASD and their “unaffected” parents demonstrated significant reduction of expression in affected subjects (Lerer et al., 2009, 2010; Ebstein et al., 2012). The therapeutic potency of all-trans retinoic acid increases CD38 expression (Ebstein et al., 2011). There are significant correlations between *CD38* expression, VABS score and IQ in humans (Riebold et al., 2011). Similar allele frequencies for the genotyped SNPs in men and women and similar correlations between plasma OXT, *CD38*, or human OXTR SNP variants and parenting behavior have been observed between human mothers and fathers (Feldman et al., 2012). However, the predictive effect of *CD38* expression was not confined to the trust-related condition. Therefore, they suggested a role of CD38 in basal OXT release rather than OXT release associated with emotional events in humans (Kiss et al., 2011); although, no direct evidence was presented.

## Subjects with Autism Spectrum Disorders Treated by Oxytocin

Indeed, positive feedback of OXT-induced OXT release was recently observed in human males. OXT was shown to enhance visual scanning of faces, particularly the eye region, as compared to a placebo. Plasma or salivary OXT levels are significantly increased after intranasal OXT administration (Andari et al., 2010; Huffmeijer et al., 2012). Nasal OXT treatment has minor effects in improving cognitive empathy and socially motivated learning (Hurlemann et al., 2010). However, nasal OXT administration facilitates trust and reduces social anxiety in conditions of social phobia and borderline personality disorder (Kosfeld et al., 2005; Bartz and Hollander, 2006; Heinrichs and Domes, 2008; Guastella et al., 2009; Guastella and MacLeod, 2012). OXT improves social cognition in autistic individuals (Domes et al., 2007; Bartz and Hollander, 2008; Guastella et al., 2010). Nasal OXT spray can modify social signals and the social feedback process in high-functioning autistic patients (Andari et al., 2010; Bartz et al., 2010; Hirosawa et al., 2012). Thus, several OXT-controlled processes have been implicated in different types of mammalian social behavior. In addition, there are three reports that indicate symptomatic improvement of male and female ASD patients with long-term OT treatment (Munesue et al., 2010; Higashida et al., 2012b; Kosaka et al., 2012).

## Conclusion

In summary, recent data suggest novel mechanisms underlying social behavior and confirm new molecular targets for pharmacological corrections of behavioral changes associated with neurodevelopmental disorders. The plasma OXT level is a reliable marker reflecting central oxytocinergic functions in humans. Rodent models are useful in this research field to investigate the molecular mechanisms underlying the disturbance of central and peripheral OXT regulation and to develop new perspectives in the therapy of human diseases characterized by social behavior deficits.

## Conflict of Interest Statement

The authors declare that the research was conducted in the absence of any commercial or financial relationships that could be construed as a potential conflict of interest.
